# Effect of parental mutuality on the quality of life of mothers of children with special health needs*

**DOI:** 10.1590/1518-8345.4385.3423

**Published:** 2021-05-21

**Authors:** Paula Rossi Baldini, Bruna Josiane de Lima, Beatriz Helena Naddaf Camilo, Juliana Coelho Pina, Aline Cristiane Cavicchioli Okido

**Affiliations:** 1 Universidade Federal de So Carlos, So Carlos, SP, Brazil.; 3 Universidade Federal de Santa Catarina, Departamento de Enfermagem, Florianpolis, SC, Brazil.

**Keywords:** Nursing, Child, Mothers, Health-related Quality of Life, Family Relations, Family, Enfermagem, Criana, Mes, Qualidade de Vida Relacionada Sade, Relaes Familiares, Famlia, Enfermera, Nio, Madres, Calidad de Vida Relacionada con la Salud, Relaciones Familiares, Familia

## Abstract

**Objective:**

to analyze the effect of parental mutuality on the quality of life related to the health of mothers who care for children with special health needs.

**Method:**

an observational, analytical and cross-sectional study with a quantitative approach. The following instruments were applied to 181 caregiving mothers: *The Medical Outcomes Study 36-Item Short Form* and *Family Management Measure* (Parental Mutuality subscale). In the statistical analysis, Spearmans correlation and univariate and multivariate linear regression were used.

**Results:**

the total score of parental mutuality was 30.8, indicating a satisfactory perception of the caregiving mother about the way the couple shares decisions regarding the care of the child. In the multivariate regression analysis, parental mutuality maintained a statistically significant association with the domains of pain, social aspects and emotional limitations of quality of life related to health (p=<0.001, 0.003, 0.002), respectively.

**Conclusion:**

parental mutuality has a positive effect on some domains of quality of life related to health. It is recommended to plan actions aimed at strengthening the complicity and connection between the couple, especially in matters related to the care of the child with special health needs.

## Abstract (pt)

**Objetivo:**

analisar o efeito da mutualidade parental na qualidade de vida relacionada sade das mes cuidadoras de crianas com necessidades especiais de sade.

**Mtodo:**

estudo observacional, analtico, transversal e de abordagem quantitativa. Foram aplicados em 181 mes cuidadoras os seguintes instrumentos: *The Medical Outcomes Study 36-Item Short Form* e *Family Management Measure* (subescala Mutualidade parental). Na anlise estatstica, utilizou-se correlao de Spearman e regresso linear univariada e multivariada.

**Resultados:**

o escore total da mutualidade parental foi 30,8, indicando uma percepo satisfatria da me cuidadora sobre a forma como o casal compartilha as decises com relao ao cuidado do filho. Na anlise de regresso multivariada, a mutualidade parental manteve associao estatisticamente significante com os domnios dor, aspectos sociais e limitaes emocionais da qualidade de vida relacionada sade (p=<0,001, 0,003, 0,002), respectivamente.

**Concluso:**

a mutualidade parental exerce efeito positivo sob alguns domnios da qualidade de vida relacionada ** sade. Recomenda-se o planejamento de aes direcionadas ao fortalecimento da cumplicidade e conexo entre o casal, sobretudo nas questes relacionadas ao cuidado do filho com necessidades especiais de sade.

## Abstract (es)

**Objetivo:**

analizar el efecto de la mutualidad parental en la calidad de vida en lo que respecta a la salud de las madres cuidadoras de nios con necesidades especiales de salud.

**Mtodo:**

estudio observacional, analtico, transversal y de abordaje cuantitativo. Fueron aplicados, a 181 madres cuidadoras, los siguientes instrumentos: *The Medical Outcomes Study 36-Item Short Form* y *Family Management Measure* (subescala Mutualidad parental). En el anlisis estadstico, se utiliz correlacin de Spearman y regresin lineal univariada y multivariada.

**Resultados:**

la puntuacin total de la mutualidad parental fue de 30,8, indicando una percepcin satisfactoria de la madre cuidadora sobre la forma en la que la pareja comparte las decisiones respecto del cuidado de los hijos. En el anlisis de regresin multivariada, la mutualidad parental tiene una asociacin estadsticamente significativa con los dominios: dolor, aspectos sociales y limitaciones emocionales de la calidad de vida relacionada con la salud (p=<0,001, 0,003, 0,002), respectivamente.

**Conclusin:**

la mutualidad parental tiene un efecto positivo sobre algunos dominios de la calidad de vida relacionada con la salud. Se recomienda la planificacin de acciones orientadas al fortalecimiento de la complicidad y la conexin en la pareja, fundamentalmente, en cuestiones relacionadas con el cuidado del hijo con necesidades especiales de salud.

## Introduction

Children with special health needs (*Crianas com necessidades especiais de sade*, CRIANES) is a broad terminology that encompasses several health conditions that vary in complexity and comorbidities^(^ 1 ^)^. This terminology was established in 1994 in the United States of America, by a working group composed of health professionals, parents and managers^(^ 2 ^)^. The main intention of this group was to institute an inclusive terminology, capable of adding a greater number of children affected by some health need in order to favor the formulation of public policies and financial investment^(^ 2 ^)^. In Brazil, the first research study that adopted the term CRIANES dates back to 2004^(^ 3 ^)^.

In general, CRIANES have developmental, behavioral, emotional and physical chronic conditions and need more attention and monitoring of the health services in addition to what is required by other children in the same age group^(^ 2 ^)^. This is an emerging population, given the advancement of health technologies^(^ 1 ^)^, the decrease in mortality from preventable causes and the increase in chronic conditions^(^ 4 ^)^.

Among the challenges experienced, the need to provide full-time care stands out, a responsibility mostly assumed by the mother, which enhances financial difficulties, social isolation, marital dissatisfaction and feelings such as hopelessness, fatigue, fear and guilt^(^ 5 ^-^ 6 ^)^. Furthermore, such challenges can compromise the quality of life related to health (QoLRH) of these caregiving mothers as well as their ability to provide care^(^ 7 ^-^ 8 ^)^.

In this sense, according to an international research study^(^ 7 ^)^ that compared the QoLRH among mothers of children with spina bifida, mothers of healthy children, and mothers of children with cerebral palsy, the QoLRH scores of mothers of children with spina bifida were significantly lower in all aspects when compared to the mothers of healthy children. The study also pointed out that there was no statistical difference between the QoLRH of mothers of children with spina bifida and mothers of children with cerebral palsy, both children with special health needs.

Parental disharmony is also highlighted as a recurring challenge among mothers caring for CRIANES^(^ 9 ^-^ 10 ^)^. According to a study carried out with parents of children with childhood cancer, a subgroup of CRIANES, it is common for the mother to assume responsibility for medical consultations, frequent hospitalizations and the entire demand for care; in contrast, the father generally assumes financial provision. This division of tasks and the difficulty in frequently bringing the whole family together can cause the sensation of a divided family, increasing possible differences regarding the childs health, treatment, strategies and management of the condition^(^ 10 ^)^.

Thus, it is important for the couple to maintain shared perspectives on care, that is, parental mutuality is important. The term parental mutuality corresponds to the feelings of intimacy, connection and understanding between the couple regarding the childs health condition^(^ 11 ^)^; it refers to the parents ability to work collaboratively^(^ 9 ^)^. A number of studies indicate that parental mutuality reflects positively on the familys ability to deal with the challenges of the childs chronic condition^(^ 12 ^-^ 13 ^)^; and that it is associated with lower levels of maternal anxiety and depression^(^ 14 ^)^ and better psychological functioning^(^ 9 ^)^. In addition, the couples strong collaboration reduces family stress and positively influences the childs quality of life^(^ 15 ^)^.

Research studies on the influence of parental mutuality on the QoLRH of caregiving mothers are scarce in the international literature and do not exist in the national literature. In addition, the existing studies focus on the emotional and mental health aspects of QoLRH^(^ 14 ^-^ 16 ^)^. Furthermore, in most cases, they analyze specific subgroups of CRIANES, such as children with asthma, cancer or autism, for example. To this end, the development of this study is justified by the originality of the proposal, as it includes mothers caring for CRIANES, regardless of medical diagnoses, as well as exploring all the domains of QoLRH. The social relevance of this study is also augmented by directing the gaze towards the caregiving mothers, often precariously assisted by the health services and the community.

Considering the above, the present study started from the following research question: What effect does parental mutuality have on the QoLRH of mothers caring for CRIANES? To this end, the study aimed to analyze the effect of parental mutuality on the QoLRH of mothers caring for CRIANES.

## Method

A study with an observational, analytical and cross-sectional design, with a quantitative approach^(^ 17 ^)^, carried out in the outpatient facilities of a large tertiary hospital located in the inland of the state of So Paulo, between November 2018 and March 2019.

Caregiving mothers of CRIANES who met the following eligibility criteria participated in the study: being a mother, ensuring most care for CRIANES between zero and 12 years old, being over 18 years old and living with a partner. There was no exclusion criterion. It is worth noting that the age of the CRIANES followed that established by the Child and Adolescent Statute. The criterion related to living with a partner is a specific orientation of the research instrument adopted^(^ 18 ^)^.

Regarding sample calculation, the following recommendation was followed: in order to obtain an alpha significance level of 5% and a power of 80%, 10 to 15 participants are required for each factor of interest, known as ratio of cases to IVs (Independent Variables)^(^ 19 ^)^, resulting in a minimum number of 90 participants.

For the production of the empirical material, contact was initially established with those responsible for the institution in order to explain about the project and request authorization for the development of the research. The data collection team consisted of three researchers, two undergraduate nursing students and a postgraduate nurse, all previously trained for the approach and for applying the instruments. After ethical appraisal, the data collection team went to the service in order to recognize the work space and dynamics. Such a strategy was necessary because it was the researchers first contact with the research scenario; they had no previous link with the team, nor with the caregiving mothers and CRIANES.

The search for potential participants was intentional and occurred in the outpatient waiting room. It was sought to recruit caregivers on different days and times in order to avoid selection bias, given that each medical specialty serves on a certain day of the week and period. Initially, the objectives of the study were explained and the mothers were invited to participate. Those who accepted the invitation received a free and informed consent form (FICF) for reading and discussion. When the caregiver did not know how to read, the companion or the researcher read aloud. The application of the research instruments lasted approximately 30 minutes and took place in the waiting room itself, as the caregiving mothers reported a concern about missing the medical consultation if they left the doctors office door.

It is worth mentioning that the researchers previously certified the participants eligibility by asking about their age and marital status. As it is an institution specialized in the treatment of children with different medical diagnoses and chronic conditions, no specific screening instrument was used in relation to children; however, the researchers were instructed to pay attention to the situations of first care. Such attention is justified because the child could be in the investigation phase of the clinical condition and, consequently, not having any special health needs, such as dependence on medications, functional limitations or use of health services beyond what is expected for a healthy child in the same age group^(^ 20 ^)^.

The following instruments were applied: characterization instrument, The Medical Outcomes Study 36-Item Short Form (SF36)^(^ 21 ^)^ and Family Management Measure FaMM^(^ 18 ^)^. The characterization instrument consisted of questions related to the socio-economic context of the caregiving mother (age, schooling, occupation and family income), as well as issues related to the CRIANES (age and clinical condition). The questions directed to the clinical condition of the CRIANES had as main objective to identify the care demanded by them, according to the following classification: demand for medication care (continuous use of medication); demand for developmental care (children require monitoring by professionals such as physiotherapists, occupational therapists, among others); demand for technological care (makes use of a technological device to maintain physiological functions, such as bladder catheterization, for example); demand for modified habitual care (requires care that differs from that offered to a healthy child in the same age group); demand for mixed care (when the child presents two or more of the above demands)^(^ 3 ^)^. This is a characterization instrument that has already been successfully applied in other studies by the research group and it is not necessary to test it previously.

SF-36 is a generic instrument for assessing QoLRH consisting of 36 items that are subdivided into eight domains: functional capacity (10 items), physical aspects (4 items), pain (2 items), general health status (5 items), vitality (4 items), social aspects (2 items), emotional aspects (3 items), mental health (5 items) and a question of comparative assessment between the current health status and that of a year ago. For each domain, a final result is calculated on a scale from zero to 100, where zero is worse health and 100 is better health. This instrument was translated and validated for Brazilian Portuguese by Ciconelli^(^ 21 ^)^.

In its entirety, the FaMM consists of 53 items and its general objective is to understand how families manage a childs chronic disease and how they incorporate this condition in the familys daily life^(^ 18 ^)^. In the present study, only the parental mutuality subscale was applied, composed of eight items which address the perception of support, sharing of opinion and satisfaction with the way the couple handles child care. The answer options for each item range from one to five, with one corresponding to I strongly disagree and five to I strongly agree. The total score of this subscale ranges from 8 to 40, with higher scores indicating greater satisfaction with the way parents handle the care of their child. The FaMM was culturally adapted to Brazil by Ichikawa and collaborators, reaching a satisfactory level of internal reliability of the items (0.7908 in the parental mutuality subscale)^(^ 18 ^)^. All the subscales that compose it can be applied to any member of the family; they are not specific to the mother figure. However, given the recruitment strategy adopted in the present study, this was the viable cut because, in general, the partner does not accompany the mother and child in outpatient care.

The data collected were coded according to the guidelines of each instrument and entered in a formatted database in the Excel spreadsheet editor. Then, the data was transferred to The SAS System for Windows (Statistical Analysis System), version 9.2.

Although the instruments reached satisfactory levels of internal consistency during their respective validation processes, above 0.70^(^ 18 ^-^ 21 ^)^, prior to the analyses, the internal consistency coefficient (Cronbachs a) was recalculated in order to certify their reliability after application in this specific population. Thus, the instruments showed high internal consistency (>0.70) when applied to mothers caring for CRIANES. This is not a mandatory stage; however, its adoption reinforces the methodological rigor of the study.

Subsequently, the Shapiro-Wilk and Kolmogorov-Smirnov normality tests were performed, verifying the absence of normal distribution of the variables. In the descriptive analysis, the categorical variables were described based on absolute (n) and relative (%) frequencies and, for the numerical variables, measures of central tendency, variability and position were used. In the analytical stage, the Spearmans correlation coefficient between the scores of the QoLRH domains and the total score of the parental mutuality was calculated. The interpretation of the correlation coefficients adopted the following classification: correlation coefficients < 0.4 (weak magnitude), > 0.4 to < 0.5 (moderate magnitude) and > 0.5 (strong magnitude)^(^ 14 ^)^. Then, univariate and multivariate linear regression analysis was performed using the Stepwise criterion for variable selection. For the tests, a significance level of 5% was adopted^(^ 19 ^)^.

The development of the study complied with the national and international standards of ethics in research involving human beings and was approved by the Research Ethics Committee of the Federal University of So Carlos - CAAE: 91091318.9.0000.5504 under opinion number: 2,735,827. Subsequently, it was approved by the Research Ethics Committee of the Clinical Hospital of the Ribeiro Preto Medical School, USP - CAAE: 91091318.9.3001.5440 under opinion number: 2,748,531.

## Results

The study included 181 caregivers of CRIANES with a mean age of 31.5 years old. With regard to occupation and source of income, 108 (59.7%) did not perform any paid work and were housewives, with a mean family income of 1,949 reais. As for schooling, 108 (60%) had completed high school or higher education, while 31 (17%) reported not having completed elementary school.

As for the CRIANES, their mean age was 3.2 years old, standard deviation 3.62, minimum zero, median 6, and maximum 11. Regarding the demands for care, 151 (83%) used medications continuously; 40 (22%) were using some type of technological device, such as a gastric feeding tube; 64 (35%) had a modified habitual care demand, such as saturation monitoring or thickener diet, among others; and 90 (49.7%) children were regularly monitored by other health professionals such as physiotherapists, phonoaudiologists, and occupational therapists, among others. It is important to note that many of these CRIANES had more than one demand for care at the same time.

With regard to the QoLRH of these caregivers, when asked to rate health in general, currently compared to a year ago, 96 (53%) caregivers answered that they were slightly better now than a year ago, while seven (3.9%) responded that they are much worse now than they were a year ago. Among the eight domains of QoLRH, vitality, emotional aspects and mental health were the domains with the lowest medians (55, 66.7 and 68, respectively). The domains that presented the highest medians were physical aspects (100) and functional capacity (95).

The median of the total score of parental mutuality was 33 (score ranging from 8 to 40), therefore indicating a satisfactory perception of how the couple shares decisions regarding child care. Table 1 presents a detailed description of the eight domains of QoLRH and parental mutuality.

**Table 1:** Description of the scores of the QoLRH and parental mutuality domains according to mean, standard deviation, minimum value, maximum value, median and quartiles. Ribeiro Preto, SP, Brazil, 2018-2019

QoLRH domains	Mean	S.D.	Min.	Q1	Median	Q3	Max.
Functional capacity	87.5	18.2	10	80	95	100	100
Physical aspects	70.0	39.5	0	50	100	100	100
Pain	66.7	28.2	0	51	72	100	100
General health status	70.1	22.4	5	52	75	92	100
Vitality	53.2	25.0	0	30	55	75	100
Social aspects	76.4	22.6	12.5	62.5	75	100	100
Emotional aspects	56.1	46.2	0	0	66.7	100	100
Mental health	64.8	24.7	0	48	68	84	100
Parental mutuality	30.8	6.82	11	28	33	36	40

The correlation matrix involving the parental mutuality score and the scores for the eight QoLRH domains is shown in Table 2. In this table, positive correlations of low magnitude and statistically significant are observed between parental mutuality and seven of the QoLRH domains, except the vitality domain.

**Table 2:** Spearman's correlation between parental mutuality and QoLRH domains. Ribeiro Preto, SP, Brazil, 2018-2019

		Functional capacity	Physical Limitations	Emotional Limitations	Pain	Mental health	Vitality	Social Aspects	General Health Status
Parental mutuality	r* = p^[Table-fn TFN2]^ =	0.15773 0.034	0.20807 0.0049	0.22532 0.0023	0.32222 <0.0001	0.16131 0.0301	0.13225 0.076	0.2646 0.0003	0.16159 0.0298

* r = Spearman's correlation coefficient;

p = p-value

Univariate linear regression analysis was also used to separately study the relation of parental mutuality with the scores of the QoLRH domains, as shown in Table 3.

**Table 3:** Effect of parental mutuality on the scores of the QoLRH domains, according to the univariate linear regression model. Ribeiro Preto, SP, Brazil, 2018-2019

	Variable	Beta* (SE) ^[Table-fn TFN4]^	p-value	R^2[Table-fn TFN5]^
Parental mutuality	Functional capacity	0.15 (0.07)	0.034	0.0249
	Physical Limitations	0.19 (0.07)	0.005	0.0433
	Emotional Limitations	0.21 (0.07)	0.002	0.0508
	Pain	0.32 (0.07)	<0.001	0.1038
	Mental health	0.16 (0.07)	0.030	0.0260
	Vitality	0.13 (0.08)	0.076	0.0175
	Social Aspects	0.26 (0.07)	<0.001	0.0700
	General Health Status	0.16 (0.07)	0.030	0.0261

* Beta = Regression coefficient;

SE = Beta Standard Error;

R^2^= Coefficient of determination. Variables without normal distribution were transformed into posts/*ranks*

According to Table 3, parental mutuality had a statistically significant effect in seven domains of QoLRH, except in the vitality domain, a result similar to that found in Spearmans correlation. Furthermore, according to the coefficient of determination (R^2^), it can be said that parental mutuality contributes 10.3% to the variation in the score of the pain domain.

For that, the seven domains entered the multivariate linear regression model using the Stepwise Backward Wald method. Table 4 shows the statistically significant relations maintained in the multivariate linear regression model. In general, the caregivers who had high scores for the QoLRH domains (emotional limitations, pain and social aspects) were those with the highest parental mutuality score. In other words, the caregivers who indicated a satisfactory perception of how the couple shares decisions regarding the care of the child were those who presented fewer impairments in their daily activities due to pain or emotional changes as well as those who indicated little interference of the childs health condition in their social activities.

**Table 4:** Effect of parental mutuality on the scores of the QoLRH domains, according to the multivariate regression model. Ribeiro Preto, SP, Brazil, 2018-2019

	Variable	Beta* (SE) ^[Table-fn TFN7]^	p-value	R^2[Table-fn TFN8]^
Parental mutuality	Emotional Limitations	0.21 (0.07)	0,002	0.0508
	Pain	0.26 (0.07)	<0.001	0.1038
	Social Aspects	0.21 (0.07)	0,003	0.0440

* Beta = Regression coefficient;

SE = Beta standard error;

R^2^= Coefficient of determination

## Discussion

With regard to QoLRH scores, vitality and emotional aspects were the domains with the lowest medians (55 and 66.7, respectively). When comparing this result with the Iranian study already contextualized in the introduction, it is observed that the Iranian mothers of children with spina bifida obtained lower mean values, being 27.7 for emotional aspects and 40.4 for vitality^(^ 7 ^)^. It is also important to highlight a study carried out in the State of Minas Gerais that aimed to evaluate the effect of a resistance exercise program on the QoLRH of mothers of children and adolescents with cerebral palsy who, from the application of SF-36, identified a median for the vitality domain identical to that presented in this research (55.0); however, after the intervention with physical exercise, the median of the vitality domain increased to 77.5^(^ 8 ^)^.

According to the results, the parental mutuality score was 30.8 (score ranging from 8 to 40), indicating a satisfactory perception of how the couple shares decisions regarding child care. This result corroborates with the study that validated the FaMM for the Brazilian culture, in which the participants were 262 family members of children aged 1 to 17 years old with chronic conditions. According to the authors, the mutuality dimension of the parents reached the highest mean among the other dimensions of the instrument with a score of 77.6 (score ranging from 20 to 100)^(^ 18 ^)^.

In addition, a recent international study that applied the FaMM to 142 parents of children with asthma identified a mean parental mutuality score close to the present study (32.14) and an intimate relation with asthma control^(^ 13 ^)^. According to the authors conclusions, the families that worked together to deal with the challenges associated with asthma had fewer difficulties in family life and, consequently, better control of childhood asthma. The study reinforces the importance of offering support and care to the family in order to favor reciprocity between the couple^(^ 13 ^)^.

On the other hand, despite the fact that the caregivers have pointed out positive perceptions regarding parental mutuality, there are studies in the literature with qualitative approaches where caregivers of CRIANES claim to feel lonely and devalued and reinforce the desire to share care and to feel safe^(^ 3 ^,^ 22 ^)^. In the same perspective, a study with a quantitative approach carried out with 100 caregiving mothers of CRIANES identified that, although most of them were married or in a stable relationship, this condition did not influence the reduction of their physical, emotional and social burden^(^ 6 ^)^. On the other hand, a statistically significant association was observed between marital status and family risk, that is, caregiving mothers who had the presence of a partner had a reduced family risk and less social vulnerability^(^ 23 ^)^.

In the multivariate regression model, parental mutuality showed a statistically significant relationship with the emotional limitations domain of QoLRH (p=0.002), that is, caregivers with a better perception of parental mutuality showed less impairments in their daily activities due to emotional issues. In this sense, this result can be supported by a Portuguese study carried out with 201 parents of children with cancer, whose main hypothesis was that parental mutuality was related to lower levels of anxiety and depression^(^ 14 ^)^. After analyzing the data, the hypothesis was confirmed as parental mutuality was negatively correlated with anxiety and depression (p=<0.001). The authors suggested that, when parents work in a supportive and collaborative manner to manage their childrens condition, better psychological functioning is observed.

In the same direction, the findings of a research study that recruited 263 parents of children with asthma, diabetes, obesity and epilepsy seen at three hospitals in Portugal also coincide^(^ 9 ^)^. According to this study, the involvement of the fathers was associated with a more favorable status in multiple dimensions of the maternal psychological functioning. Parental mutuality also proved to be relevant among couples who have children with epilepsy, reducing the stress levels of both; in addition, mutual understanding strengthened the parents confidence to manage the crises of the child with epilepsy^(^ 15 ^)^.

In addition, the multivariate regression model suggests that parental mutuality had a positive effect on the pain domain of QoLRH (p=<0.001), that is, caregivers with a better perception of parental mutuality had fewer impairments in their daily activities due to pain. In order to discuss this finding, the starting point was the perspective that emotional problems can make caregivers more likely to report problems such as headache, stomach pain and recent pain experience^(^ 24 ^)^. For that, the aspects discussed in the previous paragraphs can also support this finding.

On the other hand, pain complaints can be directly related to the provision of full-time care. According to the results of a study carried out with 177 mothers of children with some type of disability that aimed to analyze how much the childs ability to walk is related to the presence of musculoskeletal pain, including the frequency and severity of pain on the mothers back, shoulders and elbows, significantly increased as the childs level of walking decreased (p<0.05)^(^ 25 ^)^. Therefore, the present research is based on the perspective that, when the couple lives a synchronized experience, the provision of care is shared, minimizing possible pain resulting from physical effort.

In the multivariate regression model, parental mutuality also showed a statistically significant relation with the social aspects domain of QoLRH (p=0.003) suggesting that caregivers who maintain shared perspectives with their partners suffer less interference from the childs health condition in their social activities. This finding becomes relevant as the reduction of family interactions and recreational activities is common among CRIANES caregivers^(^ 9 ^,^ 26 ^)^.

In addition, in the present research, approximately 60% of the caregivers did not perform any paid work and were housewives, an aspect that contributes to social distancing. In this sense, a survey conducted in the USA that compared labor changes among couples who had or did not have a child classified as CRIANES indicated that both mothers and fathers of CRIANES suffer from lack of work when compared to those couples who do not have CRIANES^(^ 26 ^)^. The authors also suggest that work provides a break for mothers of CRIANES, resulting in better mental health and less social impact.

In the same direction, according to a study that analyzed the mothers perception about paternal care for children/adolescents with chronic diseases, when the woman has a formal job, the partner has more time to participate in the care activities^(^ 27 ^)^. An example of sharing and reciprocity between the couple was presented in a research study that described the family dynamics of families of children that required multiple, complex and continuous care^(^ 28 ^)^. In this case, the child remained only at home, did not attend schools or nurseries due to seizure episodes that were difficult to control, but the couple rearranged themselves in order to maintain the childs care as a priority without any of them having to leave paid work. Thus, during the day the mother worked and the father assumed the care responsibilities; at night, the couple reversed their functions^(^ 28 ^)^.

With regard to the limitations of this study, its cross-sectional approach stands out, which makes it impossible to establish cause and effect relationships. In addition, not including the partners perception regarding parental mutuality is considered a limitation of the study. In this sense, new studies are suggested with a longitudinal design and expanded participation of the couple.

Finally, this research contributes to the advancement of scientific knowledge for the health and nursing area as it presents new elements that support the relevance of family interventions aimed at promoting shared coping between the couple and, consequently, improving the quality of life of the caregiving mothers.

## Conclusion

It is concluded that the results presented met the objective and answered the research question. From the statistical analysis, it is suggested that parental mutuality has a positive impact on three domains of QoLRH, namely: pain, emotional limitations and social aspects. In view of the relevance of the effect of parental mutuality on the QoLRH of mothers who care for CRIANES, it is recommended that the health professionals pay attention to the planning of actions aimed at strengthening complicity and connection between the couple, especially in matters related to the care of children with special health needs.
